# Tie2-expressing monocytes/macrophages promote cerebral revascularization in peri-infarct lesions upon ischemic insult

**DOI:** 10.1038/s41392-021-00637-w

**Published:** 2021-08-09

**Authors:** Yuqiao Sheng, Xixi Duan, Yanru Liu, Feng Li, Shengli Ma, Xiaoping Shang, Xiao Wang, Yangyang Liu, Rui Xue, Zhihai Qin

**Affiliations:** 1 Medical Research Center, The First Affiliated Hospital of Zhengzhou University, Zhengzhou, Henan China; 2 Department of Neurology, The First Affiliated Hospital of Zhengzhou University, Zhengzhou, Henan China; 3 Biotherapy Center, The First Affiliated Hospital of Zhengzhou University, Zhengzhou, Henan China; 4 Department of Emergency, The First Affiliated Hospital of Zhengzhou University, Zhengzhou, Henan China; 5 Department of Medical Records, The First Affiliated Hospital of Zhengzhou University, Zhengzhou, Henan China; 6 Department of Magnetic Resonance, The First Affiliated Hospital of Zhengzhou University, Zhengzhou, Henan China

**Keywords:** Experimental models of disease, Cardiovascular diseases

**Dear Editor,**

Strokes cause 5.8 million deaths each year. Among these victims, ~30% are from China^1^. Acute ischemic stroke (AIS) is the most prevalent subtype of strokes. Although drugs can alleviate the symptoms, the recoveries of functional vessels within ischemic areas are the critical factor determining the prognosis of patients suffering from AIS^2^. Nevertheless, the mechanisms involved in cerebral revascularization remain largely unknown. Myeloid cells are among the first cells arriving around and within the injured areas after the ischemic assault^3^. A specific subgroup of Tie2-expressing monocytes (TEMs) has demonstrated vessel-repairing properties in tumors and ischemic limbs^4,5^. But, it is not clear whether TEMs participate in revascularization and neurological recovery in AIS. Hence, we explore the impacts of TEMs on the prognosis of AIS and the potential mechanism beneath with clinical samples and mouse models.

Pre-therapeutic blood samples from patients within 24 h after onset of AIS and from age-matched controls (AMCs) were collected and analyzed to determine whether TEMs are upregulated in response to ischemic brain injury. The demographics of the enrolled participants are listed in Supplementary Table 1. Figure1a shows representative magnetic resonance imaging (MRI) from an enrolled patient taken before emergency treatment at the onset day. By flow cytometric analysis, we found the proportion of circulating monocytes relative to the total number of white blood cells in patients with AIS was higher than in the AMCs (Supplementary Figs. S1, 2). Similarly, the proportion and level of Tie2 expressing in CD14^+^ monocytes were higher in the AIS group than in AMC (Fig. 1b). Within 24 h before the patient was discharged, the modified Rankin Scale (mRS) was used to estimate stroke prognosis (scores ≤2 consider better clinical outcome). Although the Tie2 expression was not correlated with the accurate mRS score of AIS patients (Supplementary Fig. S2d), patients with mRS scores ≤2 presented higher Tie2 expression in CD14^+^ monocytes than scores >2 at baseline evaluation (Fig. 1b). Overall, our clinical study demonstrated the frequency of TEMs (CD45^+^/CD14^+^/Tie2^+^) in circulation positively related to AIS patients’ good prognosis as assessed by mRS score.

**Fig. 1:**
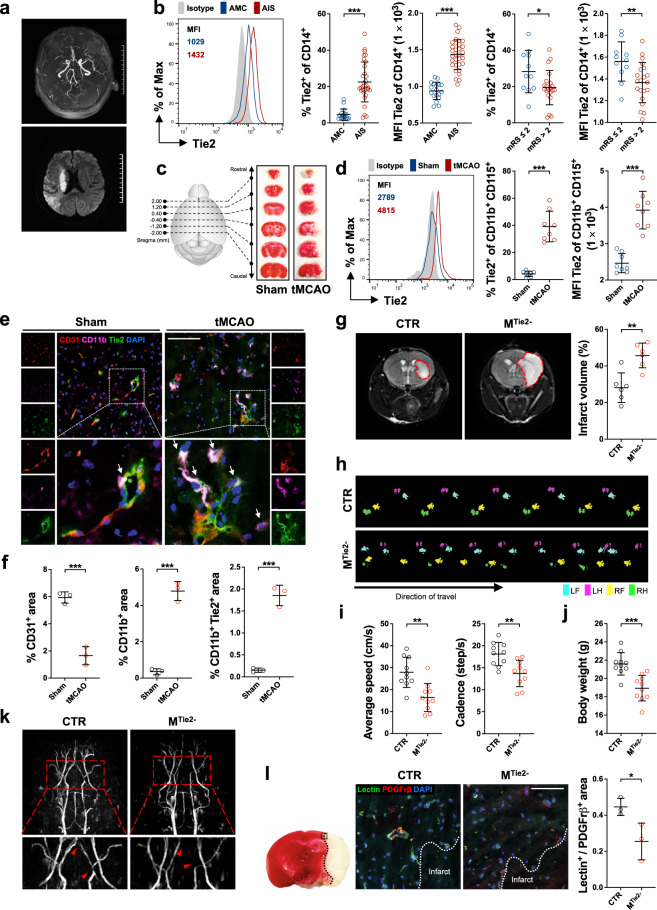
Tie2-expressing monocytes/macrophages promote revascularization in ischemic brain. **a** Representative magnetic resonance imaging (MRI) scans of a recruited patient taken with magnetic resonance angiography (MRA; upper) and coronal high-b-value diffusion-weighted imaging (below), showing severe stenosis and occlusion of the right middle artery of the patient and extensive white matter hyperintensities. **b** Flow cytometric analysis of blood samples from 32 acute ischemic stroke (AIS) patients and 18 age-matched controls (AMCs). A representative histogram plot of Tie2 staining in circulating CD14^+^ monocytes is determined with mean fluorescence intensity (MFI) and statistical analysis of Tie2 expression in AIS patients compare with AMCs. Statistics in AIS outcomes by mRS scores, ≤2 (11 patients) vs. >2 (21 patients). Each dot in the plots corresponds to one participant. **c** Representative 2,3,5-triphenyltetrazolium chloride (TTC) staining images from sham- and transient middle cerebral artery occlusion (tMCAO)-treated mice. **d** Tie2 expression in CD11b^+^/CD115^+^ circulating monocytes was analyzed by flow cytometry in Sham and tMCAO groups mice at 24 h post surgery. Each dot in the plots corresponds to one mouse (*n* = 8 per group). **e** Immunofluorescence staining in the brain ischemic area of tMCAO and sham mice at 24 h after surgery. CD11b^+^/Tie2^+^ TEM cells (arrowheads) surround the remaining CD31^+^ endothelial cells. Scale bars, 100 µm. **f** Morphometric analysis of TEMs in the cerebral obstruction zone of tMCAO and sham mice with Caliper InForm software. Each dot in the plots corresponds to one mouse (*n* = 3 per group). **g** Typical T2-weighted images of the cerebral lesion at bregma from M^Tie2−^ and CTR mice at 24 h post tMCAO. Statistical analyses showed quantitative results of MRI. Each dot in the plots corresponds to one mouse (*n* = 6 per group). **h** Gait status of M^Tie2−^ and CTR mice was assessed at 7 days after tMCAO using the CatWalk system. (LF: left front paw; LH: left hind paw; RF: Right front paw; RH: right hind paw). The average speed, cadence (**i**), and bodyweight (**j**) of both M^Tie2−^ and CTR mice were recorded, and mean values were calculated at 7 days after tMCAO (*n* = 10 per group). **k** MRA was applied to observe the status of functional vessels in mice 24 h post tMCAO. Scans in M^Tie2−^ mice showed more obvious occlusion and stenosis in the right artery (arrowheads) compared with CTR. **l** The boxed area in the TTC-stained brain section indicates the area where representative photomicrographs were taken. Double immunostaining of Lectin (green) with PDGFrβ (red) in the peri-infarct area of the mouse brain from CTR and M^Tie2−^ mice at 24 h post tMCAO is shown in the merged image. Scale bars, 100 μm. The ratio of functional capillaries/pericytes was analyzed by Caliper InForm software. Each dot corresponds to one mouse (*n* = 3 per group). All data were statistically analyzed by unpaired *t* test (mean ± SD; *p < 0.05; **p < 0.01; ***p < 0.001)

Based on CD16 expression, we further divided monocytes into three main subsets: the classical subset (CD14^++^/CD16^−^), the non-classical subset (CD14^+^/CD16^++^), and the intermediate subset (CD14^++^/CD16^+^). Flow cytometric analysis showed the scales of both non-classical and intermediate monocytes were higher in patients with AIS than in AMCs, and of classical monocytes were lower accordingly (Supplementary Fig. S3a, b). Tie2 was expressed in all three subsets for AIS patients but most strongly within the intermediate one (Supplementary Fig. S3c). Intriguingly, as no statistically significant association was found between circulating CD14^++^/CD16^+^/Tie2^+^ monocyte counts and AIS patient outcomes (Supplementary Fig. S3d), and as Tie2 expression did not always correspond with the three monocyte subsets in line with other reports, we suggest the current monocyte nomenclature may inadvertently conceal the remarkable roles of the Tie2^+^ monocyte population.

To validate the clinical data, we investigated circulating monocyte accumulation and their Tie2 expression in peripheral blood samples from sham- and transient middle cerebral artery occlusion (tMCAO)-treated mice (Fig. 1c). The mouse model of tMCAO has been widely used to mimics the clinical context in patients with AIS. Compared with the sham group, Tie2 expression of the tMCAO-treated mice was remarkably upregulated at 24 h post surgery (Fig. 1d and Supplementary Fig. S4). Meanwhile, in contrast to transiently elevated in sham-treated mice, the circulating monocyte/macrophage (CD115^+^/CD11b^+^) subgroups increased significantly and stably expanded in the tMCAO group (Supplementary Fig. S4). Next, we examined whether TEM levels are raised in brain tissue after tMCAO surgery. Of note, the blood-brain barrier (BBB) formed by continuous endothelial cell (EC) membrane acts as a regulating interface with adherens and tight junctions to protect the brain compartment as an immunoprivileged site. In this scenario, questioning whether myeloid-derived TEMs can penetrate the BBB into the brain parenchyma is reasonable. Here, we collected brain sections at 24 h post tMCAO induction, and immunofluorescence staining showed severely damaged blood vessels (CD31^+^) accompanied by rapidly accumulating microglia and monocytes/macrophages (CD11b^+^) in the occlusion areas of tMCAO mice (Fig. 1e, f). Notably, emerging TEMs (CD11b^+^/Tie2^+^) were identified around ruptured blood vessels, and they were not colocalized with the fractalkine receptor CX3CR1, a highly expressed marker on mature microglia (Supplementary Fig. S5). Our data suggest TEMs infiltrate the injured brain tissue from the peripheral blood after tMCAO induction.

Next, we generated a transgenic mouse model of Tie2^flox/−^Lyz^Cre+^ mice (M^Tie2−^) and used the littermates with a genotype Tie2^flox/+^Lyz^Cre+^ as the control (CTR; Supplementary Fig. S6) to investigate the effects of TEM deficiency in the ischemic brain. As shown in Fig. 1g, Tie2 deficiency in monocytes/macrophages was associated with markedly increased infarction areas by MRI performance 24 h post tMCAO. Statistical analysis of the data from MRI screening indicated the infarct volumes were enlarged mainly in mice lacking Tie2^+^ myeloid cells (Fig. 1g), which further confirms the effects of TEMs on infarction in vivo. Consistent with brain injury levels, the stepping speed and cadence were decreased in M^Tie2−^ mice compared with the CTR counterparts at 7 days after surgery (Fig. 1h, i), indicating that Tie2 deficiency delayed functional improvement. In the meantime, bodyweight loss was more frequently observed in M^Tie2−^ mice after tMCAO induction (Fig. 1j). Taken together, these results indicate the myeloid deficiency of Tie2 promotes the development of infarction and impairs the recovery of motor function in tMCAO-treated mice.

Considering the pro-angiogenesis function of TEMs, we wondered if TEMs support recovery after AIS via revascularization. We generated lentivirus-transduced macrophages overexpressing Tie2 and performed the tube formation assay. In comparison with the vector-transduced counterparts, Tie2-overexpressing macrophages significantly enhanced tube formation of bEnd.3 mouse cerebral endothelial cells (Supplementary Fig. S7), indicating the infiltrating TEMs may initiate the reconstruction of cerebral microvessels after ischemia. We next analyze the gene expression of Tie2 agonist angiopoietin-1 and -2 in brain lysates 24 h after tMCAO. We observed the proangiogenic gene *Angpt2* was 12 times increased in the ischemic cortex versus the contralateral area, but not *Angpt1* (Supplementary Fig. S8). The magnetic resonance angiography suggested the blood supply through the right cerebral artery occluded more severely after ischemia/reperfusion in M^Tie2−^ mice in contrast to CTR in vivo (Fig. 1k). In agreement, the cerebrovascular in peri-infarct regions of M^Tie2−^ mice appeared less perfused (Lectin^+^) than in the CTR (Fig. 1l), together with the lower ratio of Lectin^+^/PDGFrβ^+^ area, strongly suggested worse vessel recovery and function in M^Tie2−^ mice. Overall, these data imply the TEMs may reinforce the resilience of the peri-infarct regions to mount and activate vessel repair processes for survival quickly.

In conclusion, we demonstrate here bone marrow-derived TEMs promote endogenous revascularization in the mouse brain after ischemic injury. More notably, in patients with AIS, the relative changes in peripheral TEM counts were significantly related to better outcomes. Our findings highlight the necessity to better understand the mechanisms of the endogenous and protective responses in the brain after ischemia/reperfusion. As such, the identified functions of TEMs may offer new therapeutic avenues for augmenting the degree of spontaneous recovery after AIS.

## Supplementary information

supplementary materials

## Data Availability

Data are available from the corresponding author upon reasonable request.
