# Supplementary material for: Tie2-expressing monocytes/macrophages promote cerebral revascularization in peri-infarct lesions upon ischemic insult
Source: Signal Transduct Target Ther. 2021 Aug 9;6:295. doi: 10.1038/s41392-021-00637-w (PMC8349906; doi:10.1038/s41392-021-00637-w)
Supplement: Supplementary file 1 — supplementary materials [file 41392_2021_637_MOESM1_ESM.docx]

Supplementary Materials for

**Tie2-expressing monocytes/macrophages promote cerebral revascularization in peri-infarct lesions upon ischemic insult**

Yuqiao Sheng^1,§,*^, Xixi Duan^1,§^, Yanru Liu^2^, Feng Li^3^, Shengli Ma^4^, Xiaoping Shang^5^, Xiao Wang^6^, Yangyang Liu^1^, Rui Xue^1,*^, Zhihai Qin^1,*^

^1^ Medical Research Center, The First Affiliated Hospital of Zhengzhou University, Zhengzhou, Henan, China, 450052

^2^ Department of neurology, The First Affiliated Hospital of Zhengzhou University, Zhengzhou, Henan, China, 450052

^3^ Biotherapy Center, The First Affiliated Hospital of Zhengzhou University, Zhengzhou, Henan, China, 450052

^4^ Department of Emergency, The First Affiliated Hospital of Zhengzhou University, Zhengzhou, Henan, China, 450052

^5^ Department of Medical Records, The First Affiliated Hospital of Zhengzhou University, Zhengzhou, Henan, China, 450052

^6^ Department of Magnetic Resonance, The First Affiliated Hospital of Zhengzhou University, Zhengzhou, Henan, China, 450052

^§^ These authors contributed equally to this work

**Corresponding Authors**

* These authors share senior authorship; to whom should be addressed: zhihai@ibp.ac.cn (Z.Q.), xuerui04301617@126.com (R.X.), phoebesheng@hotmail.com (Y.S.)

**This Supplementary file includes:**

Materials and Methods

Figures. S1 to S8

Tables S1

**Materials and Methods**

*Patients*

The acute ischemic stroke (AIS) patients were recruited within 24 h after onset of stroke, and age-matched controls (AMCs) were also included for comparison. All AIS patients met the criteria of the 2019 edition of the Guidelines for the Diagnosis and Treatment of Acute Ischemic Stroke in China. For current analysis, we recruited only individuals for whom a high-quality and adequately interpreted magnetic resonance imaging (MRI) examination containing at least diffusion-weighted imaging (DWI) and magnetic resonance angiography (MRA) images had been performed. Patients with a diagnosed history of malignancy or chronic renal failure as well as those taking steroids were excluded from the study. AMCs were volunteers without clinical evidence of cardiovascular or cerebrovascular disease. The ethics committee of The First Affiliated Hospital of Zhengzhou University approved this study (2019-KY-396). All patients and volunteers provided written informed consent. Clinical and pathologic data from all participants were entered and maintained in our prospective database.

*MRI*

All the enrolled AIS patients underwent advanced head MRI examinations, including DWI and MRA, at The First Affiliated Hospital of Zhengzhou University before emergency treatment on the day of admission. Whole-brain MRI was performed utilizing a SIEMENS Prisma 3.0T superconducting magnetic resonance machine. MRI scanning parameters were as follows: slice thickness = 5 mm, inter-slice gap = 1 mm, DWI (TR/TE = 3000/82 ms, b value = 1000 s/mm^2^), and 3D-TOF-MRA (TR/TE = 22/3.5 ms). Images from 37 patients were reviewed by both an experienced neurologist and an experienced radiologist to identify enrolled AIS patients. After review, five patients were excluded due to the incomplete description of the data.

MRI of mouse brains was conducted on a 7T MRS-7017 MRI scanner (MR Solutions, Surrey, UK) with a dedicated mouse head coil under isofluorane (1–3% in O_2_) anesthesia, and respiration and temperature were continuously monitored. Mouse brain lesions were accessed by high-resolution axial T2-weighted (T2W) images using a fast spin-echo sequence, with the following parameters: TR/TE = 5000/45 ms, FOV = 25×25 mm^2^, slice thickness = 1 mm, number of averages = 4, number of excitations = 1, and matrix = 256×245. To calculating infarct volume of mice, we use a formula of

MRA was performed using a three-dimensional (3D) time-of-flight (TOF) sequence, with the following parameters: TR/TE = 25/3 ms, FOV= 25×25 mm^2^, slice thickness = 0.1 mm, number of excitations = 1, and matrix = 180×192.

*Mice*

C57BL/6 mice were purchased from Vital River Laboratories (Beijing, China). LyzCre; Tie2^-/flox^ (M^Tie2-^) and LyzCre; Tie2^+/flox^ (CTR) mice were generated previously^1^, and the parent mice used for breeding were kindly provided by Dr. Yulong He of Soochow University. Mice were housed in standard conditions of controlled temperature and lighting with ad libitum access to food and water. All experimental procedures used 6- to 8-week-old and sex-matched mice and were performed using protocols approved by the Institutional Animal Care and Use Committee of The First Affiliated Hospital of Zhengzhou University and consistent with the US National Institutes of Health (NIH) Guide for the Care and Use of Laboratory Animals (http://oacu.od.nih.gov/regs/index.htm).

*Transient middle cerebral artery occlusion (tMCAO) model and 2,3,5-triphenyltetrazolium chloride (TTC) staining*

Unilateral transient focal ischemia was induced in mice by tMCAO, following published procedures^2,3^. Mice were anesthetized by isoflurane anesthesia (maintenance 1.5–2%) and placed on a heating pad (37±0.5°C) throughout the surgery. Then make a 25G needlepoint incision on the left common carotid artery (CCA). A nylon filament suture (RWD Life Science, Shenzhen, China), 5 cm long, with a silicone rubber-coated tip (diameter 0.43 ± 0.02 mm), is advanced into the CCA cavity through the internal carotid artery toward the MCA. The distance traveled is usually 20 mm. After 60 minutes of MCAO, the suture was withdrawn, the incision was covered with cyanoacrylate glue, and the perfusion openness in CCA was verified. Cerebral blood flow (CBF) was monitored using a moorVMS-LDF1 laser Doppler monitor (Moor Instruments Ltd, Devon, UK) to confirm MCA occlusion and reperfusion. Only mice with a CBF reduction of at least 85% from the baseline during tMCAO for 60 min and CBF recovery to at least 80% of baseline after 10 min of reperfusion were included. Sham-operated mice received similar surgical procedures, except the MCA was not occluded. The animals were injected subcutaneously with 3 ml of normal saline to prevent dehydration, and the complete recovery after anesthesia was continuously monitored. Sodium penicillin was given intramuscularly to prevent postoperative infection. The selection of animals to receive tMCAO or as the sham-controls was completely randomized. For analysis of the infarction area, the mouse brain was obtained by decapitation at 24 h after reperfusion and kept at −20°C for 10 min. Coronal slices were prepared by cutting the brain tissues into six pieces with a thickness of ~1 mm. The slices were stained with 0.5% TTC (Sigma-Aldrich; Darmstadt, Germany) solution for 20 min at 37°C, fixed in 4% paraformaldehyde, and imaged.

*Flow cytometry*

Single-cell suspensions generated from peripheral blood of the mice and human study participants, as well as isolated monocytes of murine, were analyzed using flow cytometry for quantification of TEMs. Murine monocytes were identified by staining with FITC-labeled anti-CD45, APC-labeled anti-CD115, and PE/Cy5-labeled anti-CD11b mAbs, and their expression of Tie2 was quantified using PE-labeled anti-Tie2 (all from BioLegend, San Diego, CA, USA). Human monocytes were identified with fluorescein isothiocyanate (FITC)-labeled anti-CD45, phycoerythrin (PE)/Cy7-labeled anti-CD14, PE/Cy5-labeled anti-CD16, allophycocyanin (APC)-labeled anti-CD56, APC/Cy7-labeled anti-CD3, and Alexa Fluor 700-labeled anti-CD19 mAbs (all from BioLegend). Their Tie2 expression was quantified with PE-labeled anti-Tie2 (R&D Systems, Minneapolis, MN, USA). Dead cells were excluded using DAPI staining (Boster Biological Technology, Pleasanton, CA, USA). The cells were analyzed on a BD FACSVerse flow cytometer (BD Biosciences, San Jose, CA, USA) with FlowJo software (BD Biosciences).

*Lentiviral vector construction and cell transduction*

The green fluorescent protein (GFP)-expressing pCDH lentiviral vector (System Biosciences, Mountain View, CA, USA) with or without Tie2 were constructed. The cDNA for Tie2 was amplified by PCR from a cDNA library of mouse heart cells with the following primers: forward 5’-ATAGGCTAGCATGGACTCTTTAGCCGGCTTAG-3’, reverse 5’-GAATTCTCGAGTTATCCGAGGTCTGCAGAGGCTGGG-3’. The production and concentration of lentivirus were performed as previously reported^4^. The titers of pCDH-GFP-Tie2 were estimated by analyzing the GFP expression in transduced 293T cells by flow cytometry 3–5 days post-transduction with serial dilutions of the viral stocks. RAW264.7 cells were transduced with the lentiviruses according to the spinfection protocol to increase the transduction efficiency. Briefly, 1 day after seeding, RAW264.7 cells were mixed with the corresponding lentiviral supernatant (pCDH-GFP or pCDH-GFP-Tie2; multiplicity of infection [MOI] ~10) and then centrifuged 1000 *g* for 2 h at 32°C. Five days later, GFP^+^ RAW264.7 cells were determined by flow cytometry and further enriched to ensure purity by expansion and cell sorting twice more.

*Tube formation assay*

bEnd.3 mouse cerebral cortex endothelial cells (1×10^4^/well) were cocultured with lentivirus-transduced RAW264.7 cells (1×10^4^ or 3×10^4^/well) in a 96-well plate pre-coated with 64 μl/well of growth factor-reduced Matrigel (BD Biosciences, Billerica, MA, USA). After 2–3 h of incubation at 37°C and 5% CO_2_, the capillary tube structures were photographed under an Olympus BX51 microscope (Olympus Optical C., Tokyo, Japan). Tube length and area were quantified using National Institutes of Health (NIH) ImageJ (Bethesda, MD, USA).

*Immunofluorescence staining*

Mouse brains were dissected, post-fixed in 4% paraformaldehyde overnight, and dehydrated gradually in 10%, 20%, and 30% (w/v) sucrose in 0.01 M PBS for 24 h at 4°C. Frozen 5-μm-thick brain sections were blocked with 1% bovine serum albumin (BSA) and 1% Triton X-100 in PBS for 4 h. Then they were incubated with primary antibodies overnight at 4°C, before washing with PBS solution three times for 5 min and incubation with appropriate secondary antibodies for 2 h at room temperature. Nuclei were demarcated using DAPI staining (Electron Microscopy Sciences, Hatfield, PA, USA). The stained sections were imaged using a Vectra Polaris automated fluorescence microscopy system (Perkin Elmer, Hopkinton, MA, USA), and quantification was performed with InForm software (Perkin Elmer). The brain tissue slices of each mouse were imaged three times at different areas, and the average value was calculated.

The following primary antibodies were used: rabbit anti-CD31 (1:50) and rabbit anti-PDGFrβ (1:100) from Abcam (Cambridge, MA, USA), mouse anti-Tie2 (1:400) from Millipore, rat anti-CD11b (1:200) from BD Biosciences, rabbit anti-CX3CR1 (1:400) from Novus Biologicals (Littleton, CO, USA), and FITC-conjugated lectin (1:400) from Sigma-Aldrich (St. Louis, MO, USA). The secondary antibodies were: Alexa Fluor Plus 555 goat anti-rabbit IgG (1:400), Alexa Fluor Plus 555 goat anti-mouse IgG (1:400), Alexa Fluor Plus 647 goat anti-rabbit IgG (1:400), and Alexa Fluor 488 goat anti-rat IgG (1:400), all from Invitrogen Life Technologies (Waltham, MA, USA).

*CatWalk-assisted gait analysis*

Mouse gait analysis was conducted using the CatWalk XT Automated Gait Analysis System (Noldus Information Technology, Wageningen, The Netherlands). Mice training and CatWalk analysis were performed as previously reported^5,6^, and further technical details can be found in the Noldus Company manual (version 10.5). Briefly, before tMCAO surgery, mice were trained to cross a 1-meter-long glass walkway with a fluorescent light transmitting through the floor in a dark room. Each footfall was captured and collected by a CCD camera mounted under the runway to visualize the different paw contacts. Locomotion parameters were then automatically assessed with the CatWalk program. The mice were neurologically evaluated by two technicians who were utterly blind to the experimental group. For this study, we focus on the cadence and average speed, and the time point was 7 days after tMCAO. Data for each mouse were averaged across three compliant and continuous trials.

*Real-Time PCR*

Total RNA from mouse brain cells was extracted using the TRIzol reagent (Invitrogen Life Technologies) and reverse-transcribed into cDNA using a PrimeScript^TM^ RT reagent kit (Takara, Kusatsu, Japan). qPCR was performed on ABI StepOnePlus™ Real-Time PCR Systems (Applied Biosystems, Foster City, CA, USA) using SYBR Premix Ex Taq II (Takara). For gene expression detection in mouse brain tissue, *Hprt* was selected as the most stable reference gene according to a previous report^7^. Gene expression was measured in five biological samples, each obtained from one mouse. The following primers (Sangon Biotech, Shanghai, China) were used: mAngpt1-For CACATAGGGTGCAGCAACCA, mAngpt1-Rev CGTCGTGTTCTGGAAGAATG-A, mAngpt2-For CCTCGACTACGACGACTCAGT, mAngpt2-Rev TCTGCACCA-CATTCTGTTGGA, mHprt-For AGTGTTGGATACAGGCCAGAC, mHprt-Rev C-GTGATTCAAATCCCTGAAGT.

*Statistical analysis*

All experiments were performed at least three times independently. Data were analyzed with GraphPad Prism version 8 (GraphPad Software, La Jolla, CA, USA) and SPSS version 19.0 (IBM, Armonk, NY, USA). All quantitative data are presented as mean ± standard deviation (SD). Statistical analyses were carried out using unpaired or paired Student’s t-test, one-way or two-way ANOVA. Pearson's analysis was used to assess the correlation analysis. Differences in results were considered statistically significant at *p*<0.05. Analysis of all experimental data was conducted in a single-blind manner.

**References**

1 Chen, L. *et al.* Tie2 Expression on Macrophages Is Required for Blood Vessel Reconstruction and Tumor Relapse after Chemotherapy. *Cancer Res* **76**, 6828-6838 (2016).

2 Jackman, K., Kunz, A. & Iadecola, C. Modeling focal cerebral ischemia in vivo. *Methods Mol Biol* **793**, 195-20 (2011).

3 Lee, S. *et al.* Middle cerebral artery occlusion methods in rat versus mouse models of transient focal cerebral ischemic stroke. *Neural Regen Res* **9**, 757-758 (2014).

4 Kutner, R. H., Zhang, X. Y. & Reiser, J. Production, concentration and titration of pseudotyped HIV-1-based lentiviral vectors. *Nat Protoc* **4**, 495-505 (2009).

5 Hetze, S., Romer, C., Teufelhart, C., Meisel, A. & Engel, O. Gait analysis as a method for assessing neurological outcome in a mouse model of stroke. *J Neurosci Methods* **206**, 7-14 (2012).

6 Hamers, F. P., Koopmans, G. C. & Joosten, E. A. CatWalk-assisted gait analysis in the assessment of spinal cord injury. *J Neurotrauma* **23**, 537-548 (2006).

7 Kang, Y., Wu, Z., Cai, D. & Lu, B. Evaluation of reference genes for gene expression studies in mouse and N2a cell ischemic stroke models using quantitative real-time PCR. *BMC Neurosci* **19**, 3 (2018).

**Fig. S1 Identification of monocytes in whole blood of human.** **a** Typical forward and side scatter pattern of whole, lysed blood showing monocyte (black circle). Gating of circulating live cells was followed by exclusion of dead cells according to DAPI staining, and then CD45^+^ cells were selected as circulating leukocytes (black box). **b** CD19^+^ B cells (left, black circle), CD56^+^ natural killer cells (middle, black circle), and CD3^+^ lymphocytes (right, black circle) were gated for exclusion from analysis.


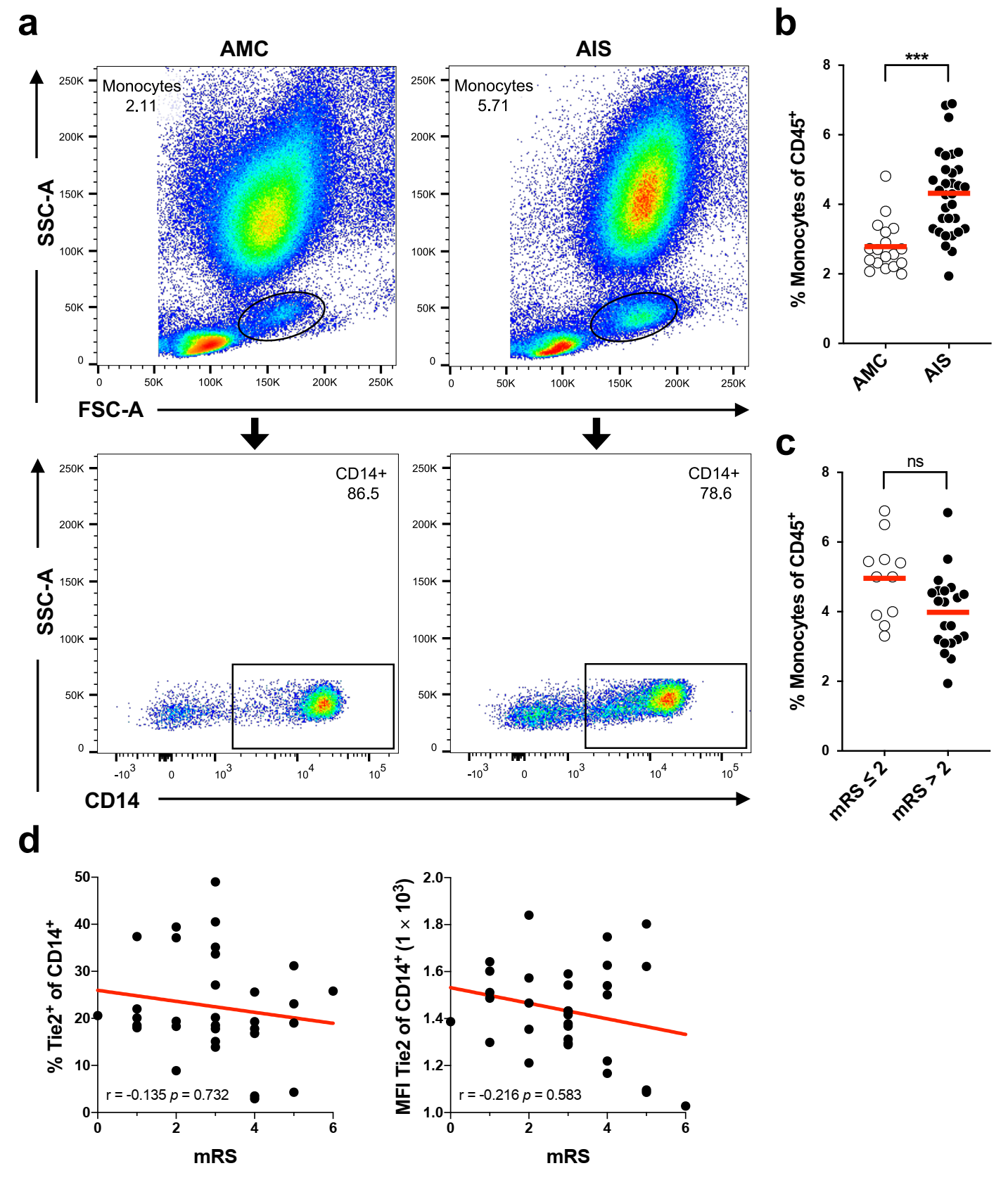


**Fig. S2 The frequency of circulating monocytes increased in response to AIS but was not related to the outcomes.** **a** Representative dot plots from flow cytometric analysis of blood samples from AIS patients and age-matched controls (AMCs). Statistical analysis of circulating monocyte frequencies of (**b**) AIS patients (n=32) vs. AMCs (n=18), and (**c**) AIS patients with mRS score ≤2 (n=11) vs. >2 (n=21). **d** Correlation analysis of Tie2 expression in CD14^+^ monocytes and the accurate mRS score of AIS patients (n=32). Each dot in the plots corresponds to one subject. Unpaired *t*-test was used for comparison between groups. (ns, not significant; ****p*<0.001).

**Fig. S3 The majority of circulating TEMs from AIS patients express CD16.** **a** Gating strategy for three monocyte subsets in samples from AIS and AMC groups based on relative CD14 and CD16 expression. Mean fluorescence intensity (MFI) of Tie2 staining in the three subsets was determined. **b** Circular distribution chart of three monocyte subsets in both AIS and AMC groups. **c** Statistical analysis showed the majority of TEMs were found within the CD14^++^/CD16^+^ monocyte subset. **d** Tie2 expression in CD14^++^/CD16^+^ monocytes was not statistically related to the mRS score of AIS patients. Each dot in the plots corresponds to one participant. Unpaired *t*-test was used for comparison between groups, and one-way analysis of variance (ANOVA) for comparison of more than three groups (ns, not significant; ***p*<0.01; ****p*<0.001).

**Fig. S4 Increased expression of Tie2 in peripheral monocytes in mice following ischemia/reperfusion injury**. **a** Gating strategy for flow cytometric analysis of peripheral blood cells. **b** Tie2 expression in CD11b^+^/CD115^+^ circulating monocytes was analyzed in two groups. D. Statistical analysis of flow cytometric results at different time points post-tMCAO surgery by unpaired *t*-test. Each dot in the plots corresponds to one mouse (mean ± SD; **p*<0.05; ***p*<0.01; ****p*<0.001).


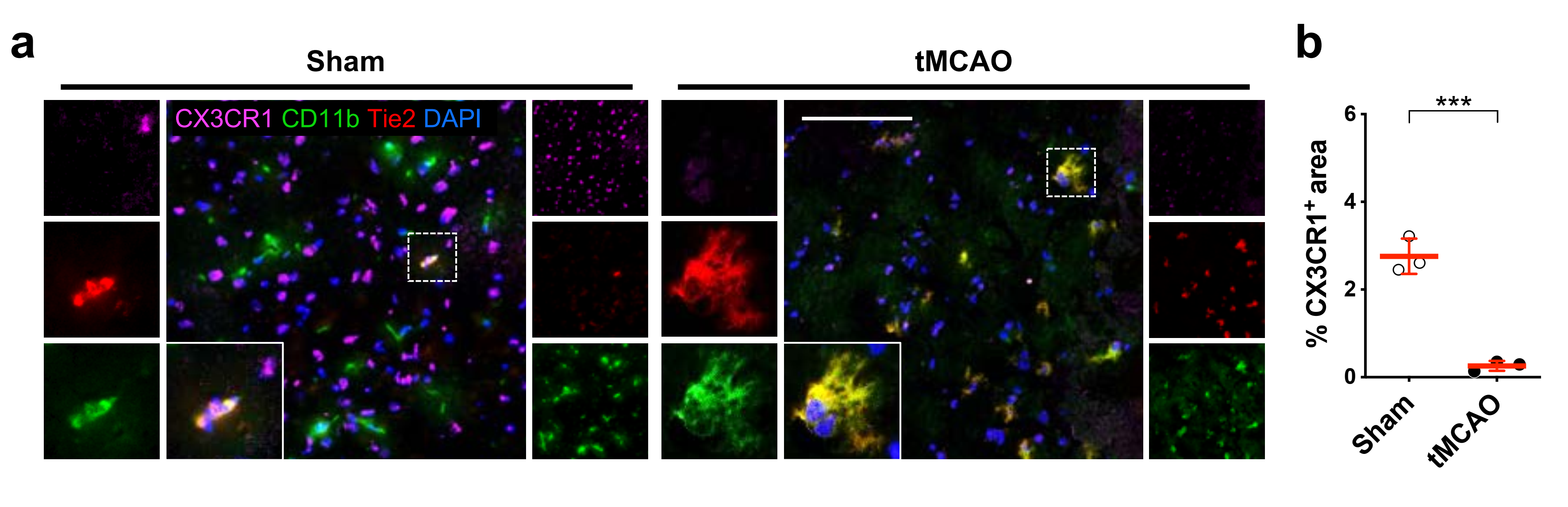


**Fig. S5 Distribution of TEMs in brain specimens of mice.** Immunofluorescence staining in the ischemic brain of tMCAO and sham mice at 24 h after surgery. **a** Representative photomicrographs show CD11b^+^/Tie2^+^ TEM cells are rarely co-stained with CX3CR1. Scale bars, 100 µm. **b** Morphometric analysis of TEMs in the cerebral obstruction zone of tMCAO and sham mice with Caliper InForm software. Each dot in the plots corresponds to one mouse (n=3 per group). Statistical analyses by unpaired *t*-test (mean ± SD; ****p*<0.001).

**Fig. S6** **Gene expression in M^Tie2-^ and CTR mice.** M^Tie2-^ (LyzCre; Tie2^-/flox^; red box) and CTR (LyzCre; Tie2^+/flox^; green box) mice were generated by breeding LyzCre^+/+^; Tie2^+/-^ mice with Tie2 ^flox/flox^ mice. The representative image shows the analysis of DNA fragments obtained from tissue lysate at the tail tip of mice born in the same litter using agarose gel electrophoresis. (M: DNA size marker; #1–13: mouse ID).

**Fig. S7** **The activity of TEMs in promoting revascularization *in vitro*.** **a** Representative images of tube formation following co-culture of 10^4^ bEnd.3 cells with Tie2-overexpressing RAW264.7 or control cells at different cell numbers (1 × 10^4^ or 3 × 10^4^). Scale bars, 200 μm. **b** Quantitative analysis of tube length and tube area. Fold changes were calculated by comparison to tubule growth of bEnd.3 cells in co-culture with RAW264.7 cells or alone. Each dot corresponds to one of three wells from one independent experiment, and experiments were performed in triplicate. Data were statistically analyzed by unpaired *t*-test (mean ± SD; ns, not significant; **p*<0.05; ****p*<0.001).


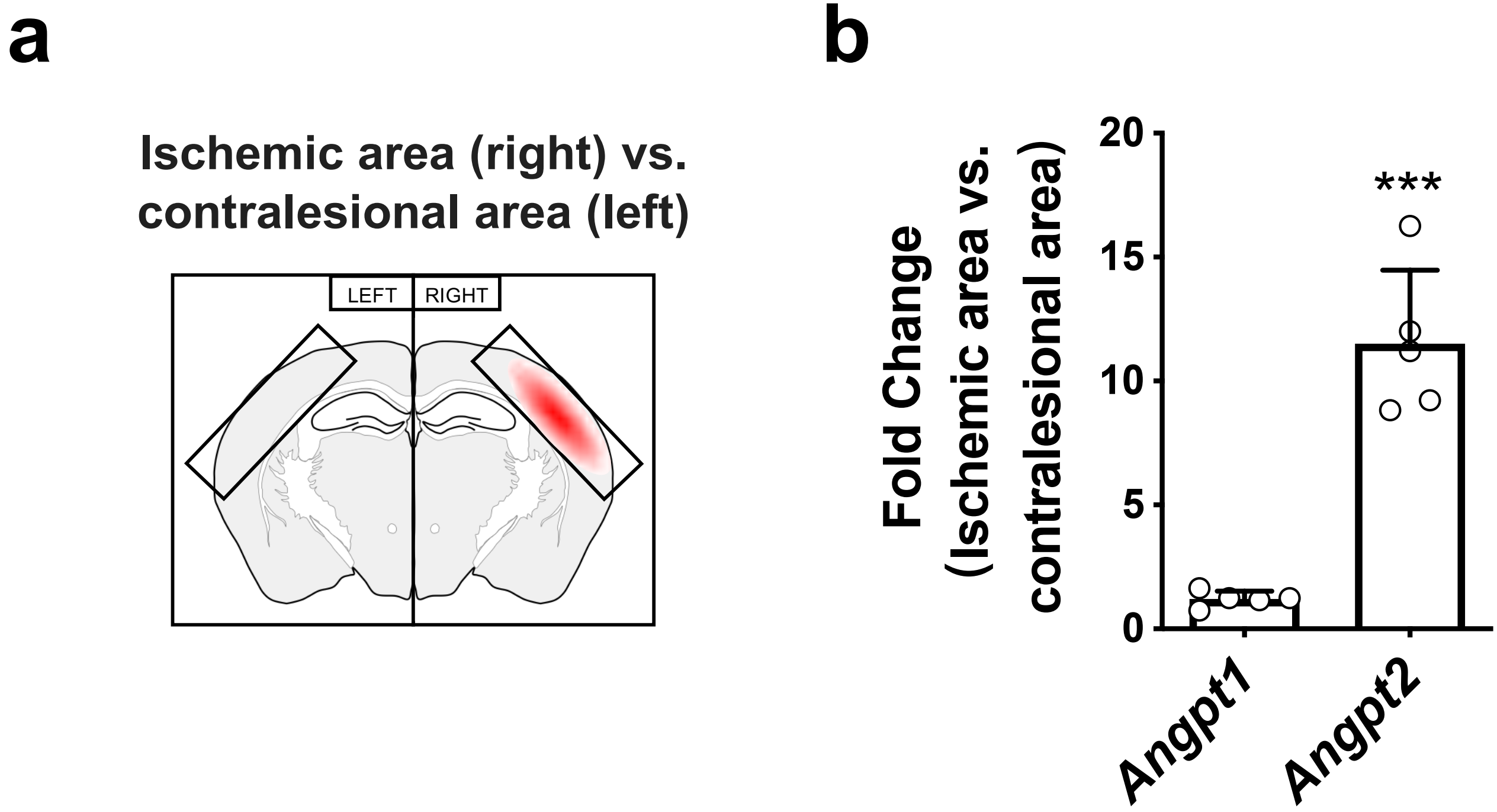


**Fig. S8** **The expression of Angiopoietin-2 upregulates in the ischemic mouse brain.** **a** Image shows the boxed area of brain regions was sampled and followed by an investigation into the molecular level of genes. **b** Comparison of gene expression by qPCR between ischemic cerebral cortex areas and contralesional areas at 24 h post-tMCAO. Mean fold-change over reference gene expression value of *Hprt*. Each group contained 5 mice. Genes differentially expressed between two regions are indicated by the asterisks. Data were statistically analyzed by paired *t*-test (mean fold change ± standard error [SE]; ****p*<0.001).

**Table S1** Demographic characteristics of AIS patients and age-matched controls (AMCs).

| **Characteristic** | **AIS patients**  **(n=32)** | **AMCs**  **(n=18)** | ***p*** |
| --- | --- | --- | --- |
| Age (years) | 67.3±17.4 | 66.4±13.2 | 0.476 |
| Sex |  |  | 0.265 |
| Male | 19(59.4%) | 10(55.6%) |  |
| Female | 13(40.6%) | 8(44.4%) |  |
| Body mass index (kg/m^2^) | 26.4±4.3 | 23.8±5.9 | 0.134 |
| Former smoker | 7(21.9%) | 5(27.8%) | 0.268 |
| Current smoker | 7(21.9%) | 4(22.2%) | 0.768 |
| Hypertension | 23(71.9%) | 9(50%) | <0.05 |
| Dyslipidemia | 22(68.7%) | 8(44.4%) | <0.05 |
| Diabetes | 7(21.9%) | 4(22.2%) | 0.153 |
| Coronary artery disease | 4(12.5%) | 2(11.1%) | 0.226 |
| Peripheral artery disease | 1(3.1%) | 0 | 0.236 |
| Atrial fibrillation or flutter | 3(9.4%) | 1(5.6%) | 0.356 |
| WBC count (×10^9^/L) | 8.55±3.21 | 6.62±2.3 | 0.245 |
| Monocytes (×10^9^/L) | 0.45±0.2 | 0.37±0.12 | 0.194 |
| Lymphocytes (×10^9^/L) | 1.48±0.57 | 1.42±0.34 | 0.612 |
| Neutrophils (×10^9^/L) | 6.355±2.87 | 4.3±1.67 | <0.05 |

WBC: white blood cell
